# Fused‐Silica 3D Chiral Metamaterials via Helium‐Assisted Microcasting Supporting Topologically Protected Twist Edge Resonances with High Mechanical Quality Factors

**DOI:** 10.1002/adma.202103205

**Published:** 2021-08-16

**Authors:** Julian Köpfler, Tobias Frenzel, Jörg Schmalian, Martin Wegener

**Affiliations:** ^1^ Institute of Applied Physics Karlsruhe Institute of Technology (KIT) 76128 Karlsruhe Germany; ^2^ Institute of Nanotechnology Karlsruhe Institute of Technology (KIT) 76021 Karlsruhe Germany; ^3^ Institute for Theoretical Condensed Matter Physics Karlsruhe Institute of Technology (KIT) 76128 Karlsruhe Germany; ^4^ Institute for Quantum Materials and Technologies Karlsruhe Institute of Technology (KIT) 76021 Karlsruhe Germany

**Keywords:** 3D chiral mechanical metamaterials, fused silica, multiphoton 3D printing, topological bandgaps, topologically protected elastic modes

## Abstract

It is predicted theoretically that a 1D diatomic chain of 3D chiral cells can support a topological bandgap that allows for translating a small time‐harmonic axial movement at one end of the chain into a resonantly enhanced large rotation of an edge state at the other end. This edge state is topologically protected such that an arbitrary mass of a mirror at the other end does not shift the eigenfrequency out of the bandgap. Herein, this complex 3D laser‐beam‐scanner microstructure is realized in fused‐silica form. A novel microcasting approach is introduced that starts from a hollow polymer cast made by standard 3D laser nanoprinting. The cast is evacuated and filled with helium, such that a highly viscous commercial glass slurry is sucked in. After UV curing and thermal debinding of the polymer, the fused‐silica glass is sintered at 1225 °C under vacuum. Detailed optical measurements reveal a mechanical quality factor of the twist‐edge resonance of 2850 at around 278 kHz resonance frequency under ambient conditions. The microcasting approach can likely be translated to many other glasses, to metals and ceramics, and to complex architectures that are not or not yet amenable to direct 3D laser printing.

## Graphical abstract

A novel helium‐assisted microcasting approach is introduced to fabricate 3D fused‐silica microstructures. For a 3D chiral fused‐silica metamaterial beam, the existence of topologically protected twist‐edge resonances with large mechanical quality factors of 2850 is demonstrated. The microcasting approach can likely be translated to many other materials, and to complex architectures that are not amenable to direct 3D laser printing.

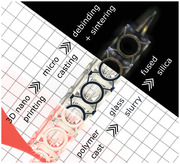

## 1 Introduction

**Figure 1:**
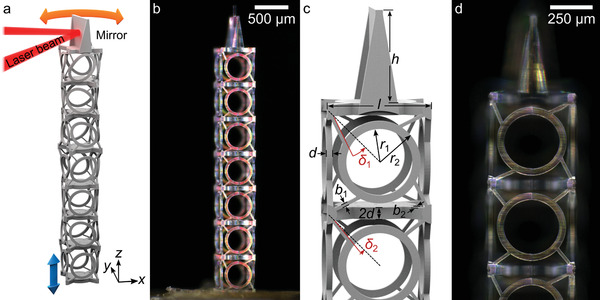
a) Scheme of the laser‐beam scanner based on a topologically protected edge mode of a diatomic chiral metamaterial beam. An axial excitation at the bottom (blue arrow) translates into a resonant twisting motion (red arrow) of the micromirror at the other end. b) Digital optical microscopy image of a fabricated sample made of fused silica. c,d) Close‐up views of the top end as‐designed (c) and as‐fabricated (d). The geometrical dimensions are listed in Table S1 (Supporting Information).

Direct multiphoton 3D printing of polymer‐based microstructures has become routine.^[^ ^1^, ^2^ ^]^ Throughout the last decade, corresponding progress has enabled numerous scientific applications in micro‐optics,^[^ ^3^, ^4^, ^5^, ^6^ ^]^ mechanics,^[^ ^7^, ^8^, ^9^ ^]^ biology,^[^ ^10^, ^11^, ^12^ ^]^ and robotics.^[^ ^13^, ^14^, ^15^, ^16^ ^]^ However, certain applications require ingredient material properties that polymers just do not provide. For example, the viscoelastic behavior of polymers implies that mechanical resonances with quality factors substantially exceeding ten are not in reach. In sharp contrast, the quality factor of quartz tuning forks, which are routinely used in watches, exceeds values of 100 000 in vacuum and 10 000 at ambient conditions.^[^ ^17^, ^18^ ^]^ For this mechanical reason as well as for optical, chemical, and durability reasons, direct 3D printing of fused‐silica structures has attracted considerable attention.^[^ ^19^, ^20^, ^21^, ^22^, ^23^ ^]^ Recent work even showed multiphoton 3D laser printing of fused‐silica microstructures.^[^ ^24^ ^]^ However, it is fair to say that presently not all interesting and relevant 3D fused‐silica microarchitectures can be manufactured along these lines with the required precision. We have encountered this limitation when working toward implementing a theoretically suggested laser‐beam scanner based on a protected edge mode in a 1D topological bandgap of a diatomic chain of 3D chiral metamaterial unit cells.^[^ ^25^ ^]^ This structure, the design of which builds on extensive previous work on topological phonons,^[^ ^26^, ^27^, ^28^, ^29^ ^]^ is illustrated in **Figure** **1**a. Therefore, we have searched for novel means of manufacturing this particular 3D microarchitecture as well as related ones in fused‐silica form.

Here, we realize such delicate 3D fused‐silica microstructures by starting with a polymer cast made by multiphoton 3D laser printing using commercially available instrumentation and photoresists. The channels in the polymer cast are evacuated, filled with helium gas, and then filled with a commercially available highly viscous slurry containing a large volume fraction of silica nanoparticles. After completed infilling and UV curing of the slurry, we thermally debind the polymer cast and the slurry's polymeric filler at 600 °C and subsequently heat the samples to temperatures up to 1225 °C under vacuum. This step sinters the silica nanoparticles, eventually forming a high‐quality solid bulk 3D silica‐glass microstructure. This procedure allows us to experimentally realize the aforementioned resonant chiral topological‐bandgap structure by fused silica. We identify the aimed‐at resonant topologically protected edge mode and measure a mechanical quality factor of 2850 under ambient conditions.

In addition to this particular novelty, the described microcasting approach can most likely be transferred from fused silica to many other glasses, to metals and ceramics, and especially also to materials that are not accessible conceptually by direct multiphoton 3D laser printing, e.g., any kind of optically opaque materials.

## 2 Microstructure Design and Fabrication

Figure 1a illustrates the targeted 1D diatomic chain of chiral unit cells with a micromirror on one end. The working principle and theoretical design has been outlined in detail previously.^[^ ^25^ ^]^ In brief, the alternation of two slightly different cubic cells (cf. Figure 1c) combined with a mirror symmetry (in the sense that the effective masses and moments of inertia of the two cells in the diatomic unit cell are the same) opens a 1D topological bandgap. This gap supports two topologically protected edge states, located on the end of the micromirror and the opposite end, respectively. Topological protection of the state on the micromirror side is crucial because it guarantees that the twist‐edge‐state frequency stays inside of the frequency interval of the 1D bandgap – regardless of the mass of the micromirror. When exciting the arrangement time‐harmonically at ultrasound frequency along the axial direction by a piezoelectric actuator, the mirror rotates back and forth (cf. Figure 1a). We verify that the topological protection persists even in the presence of strong material damping (see the Supporting Information),^[^ ^30^, ^31^, ^32^ ^]^ for example, for the low quality factors of *Q* ≈ 20 of polymeric structures (see Figure S1 in the Supporting Information). However, to obtain an appreciable resonant enhancement of the notoriously small amplitudes of piezoelectric actuators at high frequencies, low material damping, as expected for fused‐silica structures, is indispensable. The overall quality factor is determined not only by material damping, but also by air damping and anchor losses.^[^ ^18^, ^33^ ^]^ For the resonance inside of the 1D bandgap, the anchor loss is determined by the evanescent coupling of the twist‐edge state to the other end of the beam. Therefore, in principle, the mechanical quality factor related to anchor loss can be made infinitely large by making the beam infinitely long. As a practical trade‐off between overall size of the device and its mechanical quality factor, we consider seven cubic cells in Figure 1a.

**Figure 2:**
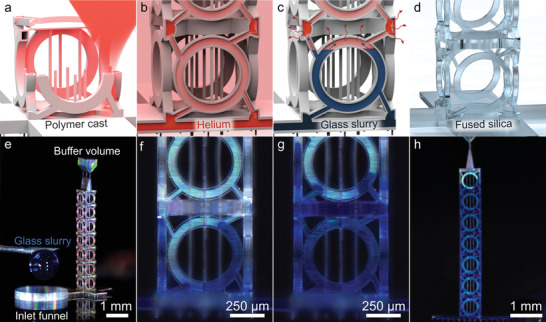
Process steps for the fabrication of 3D fused‐silica microstructures. a) Polymer casts are fabricated using multiphoton 3D printing, evacuated, and b) filled with helium gas. c) As the sample is exposed to air, the helium diffuses through the polymer cast, creates a vacuum, and thereby sucks a glass slurry into the branched channel system. d) Thermal debinding of the polymer and subsequent sintering leads to the fused‐silica 3D microstructure. e) Optical microscopy image of the fabricated cast with inlet funnel and buffer volume at the top. f–h) The blue‐colored glass slurry is gradually sucked into the channel system, as shown after 60 s (f), 150 s (g), and fills almost the entire polymer cast after 20 min (h).

Figure 1b,d shows digital optical images of a corresponding fused‐silica microstructure that has resulted from the helium‐assisted microcasting approach introduced in this work. The process steps are illustrated in **Figure** **2**. First, the polymer cast is manufactured by using a commercial 3D laser printer (Photonics Professional GT, Nanoscribe) and a commercial photoresist (IP‐S, Nanoscribe). The 3D printing parameters are given in the Experimental Section. To reduce the proximity effect, which otherwise leads to undesirable polymer cross‐linking in the channels, we do not 3D print the full complement of the targeted structure but rather only a shell around it plus some additional support beams, a bottom plate, and an inlet funnel. Second, we evacuate the developed cast and fill it with helium gas. While the sample is kept in the helium environment, we fill a droplet of glass slurry (L40, Glassomer) into the inlet funnel of the polymer cast. Directly afterward, we expose the sample to air, and the helium enclosed in the polymer cast escapes through the cast's shell. The induced vacuum sucks the viscous glass slurry into and throughout the whole polymer cast (cf. Figure 2f–h). After UV curing, we heat the samples in a tube furnace to 600 °C under ambient conditions to thermally debind both, the polymer cast and the polymeric binder contained in the glass slurry. Finally, we sinter the samples at 1225 °C under vacuum to obtain solid fused‐silica 3D microstructures. The parameters for the heat treatment are generally along the lines of previous work,^[^ ^21^ ^]^ but adapted in detail to the delicate 3D microstructures with high aspect ratio discussed here. The relative linear shrinkage of the microstructures after sintering is 22%. We find that the surface quality of the final samples is mainly determined by the cast's quality, i.e., the hatching and slicing distance used in the multiphoton 3D printing process. The measured resonance curves of various fused‐silica micro‐tuning‐forks manufactured via the presented helium‐assisted microcasting approach, shown in Figure S1 (Supporting Information), together with the corresponding finite‐element method (FEM) calculations using a linear‐elastic material with a mass density of ρ = 2.2 g cm^−3^ and a Poisson's ratio of 0.17,^[^ ^34^, ^35^ ^]^ yield a Young's modulus of 70.8 (±2.7) GPa at a frequency of around 240 kHz, which is in good agreement with the Young's modulus of 72.9 GPa of bulk fused silica.^[^ ^34^ ^]^

As verified by our targeted microstructure, the fabrication procedure works for complex branched microstructures combining small feature elements (below 16 µm width) with large overall size (around 4 mm length). Due to the polymeric scaffold, pending parts are easy to realize. The fused‐silica tuning forks shown in Figure S1 and Video S1 (Supporting Information) also demonstrate the feasibility of delicate structures with dead ends. Only for microstructures with extreme aspect ratios, associated with filling times in the range of 30 min and more, incomplete filling at dead ends can occur due to diffusion of air or evaporation of the glass slurry's solvent into the otherwise sealed cast (not depicted). For the targeted microstructure, this restriction was eased by using a buffer volume (cf. Figure 2e). Alternatively, an external overpressure can be applied gradually during the filling procedure (not used for the microstructures discussed in this work).

## 3 Results

**Figure 3:**
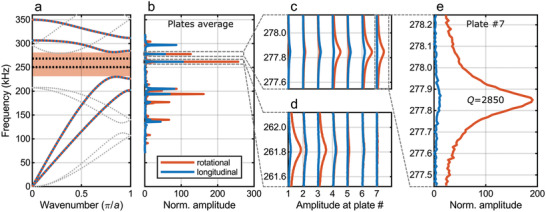
Mechanical response spectra of the 3D microstructure. a) Calculated band structure of the designed chiral metamaterial beam with topological bandgap (light‐red region) and two edge states (dashed black lines). b) Measured response spectrum, averaged over all horizontal plates of the structure (cf. Figure 1), showing both bulk and edge modes. c) Response of the individual plates for the edge mode resonance located at the top end (plate #7) and d) for the edge mode located at the bottom end (plate #1). e) The top‐end twist‐edge resonance exhibits a quality factor of *Q* ≈ 2850. In (b)–(e), the axial or longitudinal component of the displacement vector is shown in blue and the azimuthal or rotational component in red.

**Figure 4:**
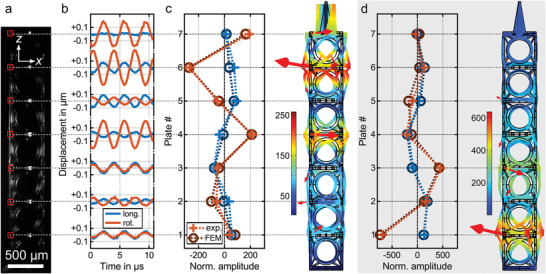
a) Side‐view optical microscopy image of the stroboscopically illuminated fused‐silica metamaterial beam. The displacements are tracked at the corner of each horizontal plate (red boxes) via optical image cross‐correlation. b) Measured time‐harmonic longitudinal (blue) and rotational (red) displacements of the individual plates at the resonance frequency *f* = 277.85 kHz of the top‐end edge mode for a longitudinal excitation at the bottom with an amplitude of 1 nm. For the rotational motion, only the in‐plane component can be captured in the form of a lateral oscillation. The rotational character was verified by additional top‐view measurements (cf. Figure S2 in the Supporting Information). c) The measured normalized displacement amplitudes at the individual plates agree well with the those of the corresponding FEM calculations (scaled by a global factor). The false‐color plot shows the local normalized displacement and the red arrows show the calculated displacement vectors at the plates’ positions. Positive (negative) amplitudes indicate an in‐phase (counterphase) oscillation with respect to the longitudinal component of plate #1. d) An analogous evaluation at *f* = 261.82 kHz reveals the mode shape of the bottom‐end edge resonance.

To show the existence of the predicted topologically protected edge resonances,^[^ ^25^ ^]^ we mount the fabricated 3D microstructures onto a piezoelectric actuator and apply a time‐harmonic axial excitation at ultrasound frequencies and amplitudes of only a few nanometers. We optically image the structure from the side as well as from the top under synchronized stroboscopic illumination using a light‐emitting diode and an ordinary complementary metal–oxide–semiconductor (CMOS) camera. This setup was used previously by us.^[^ ^9^ ^]^ We derive local displacement vectors at the corners of the microstructure's horizontal plates by means of optical‐image cross‐correlation analysis,^[^ ^36^ ^]^ which provides highly accurate displacements even for a large field of view and low magnification, respectively.^[^ ^37^ ^]^ The displacement amplitudes are normalized to the vertical excitation amplitude, which we measure at the bottom plate of the microstructure. In this manner, the properties of the glue, which is used to fix the sample to the piezoactuator, do not enter into our analysis. By sweeping the excitation frequency, we obtain the spatially resolved mechanical response spectrum of the microstructure shown in **Figure** **3**. A coarse frequency sweep from 70 to 320 kHz with frequency steps of ∆*f* = 1 kHz reveals both the bulk modes and the two modes inside of the 1D topological bandgap (cf. Figure 3a,b). In a subsequent finer frequency sampling (∆*f* = 0.01 kHz), shown in Figure 3c,d, the individual plates’ amplitudes reveal that the two resonances in the bandgap are localized at the two opposite ends of the microstructure. **Figure** **4** shows that the measured mode shapes of the two edge resonances are in good agreement with the corresponding FEM calculations of the microstructure. For the resonance at the position of the micromirror, we find a quality factor of *Q* ≈ 2850 at ambient conditions and an enhancement of the azimuthal amplitude at the top plate's edge of almost 200 with respect to the axial excitation amplitude at the bottom. The micro‐tuning‐fork measurements in Figure S1 (Supporting Information) indicate that the quality factor is not limited by material damping, but rather by air damping, and can reach values as large as *Q* ≈ 17 000 in vacuum. This value is a factor of 785 larger than that of the corresponding polymeric micro‐tuning‐forks. However, having the application as a resonant laser‐beam scanner in mind, an operation under vacuum is not desirable. Finally, we note that the calculated and measured resonances shown in Figure 3a,b, respectively, agree very well. From this agreement, we conclude that the theoretical material parameters, such as Young's modulus and mass density (see above), match those of the experiment.

## 4 Conclusion

We have introduced a helium‐assisted microcasting process based on 3D laser‐printed polymer casts filled with a glass slurry, to manufacture complex high‐quality 3D fused‐silica microstructures. As a demanding benchmark example, we realized a previously predicted chiral metamaterial beam composed of a 1D chain of 3D unit cells. Herein, we demonstrated the existence of topologically protected twist‐edge states with mechanical quality factors as large as 2850 at a frequency of 278 kHz under ambient conditions. The possibility of obtaining such high mechanical quality factors in advanced 3D microstructures might also enable the making of topological mechanical metamaterial architectures invoking 2D or 3D topological bandgaps. Furthermore, the presented microcasting process is likely transferrable to other nanocomposites and sol–gel mixtures, providing a practical and versatile approach to manufacture 3D microstructures of other constituent materials such as metals and ceramics.

## 5 Experimental Section

### Multiphoton 3D Printing

The 3D polymer casts were fabricated using the commercial instrument Photonic Professional GT (Nanoscribe, Germany) with a 25× microscope objective lens (numerical aperture (NA) = 0.8, Zeiss) and the photoresist IP‐S (Nanoscribe, Germany). The cast's nominal shell thickness was 11 µm and the footprint of the scaffold posts was 19 × 19 µm^2^. The laser power was set to 36.25 mW (back focal plane) at a scan speed of 14 cm s^−1^. The writing parameters were a slicing distance of 1.5 µm and an alternating hatching of 0.5 µm, with stitching of 347 × 347 × 223 µm^3^ blocks. The samples were printed onto indium tin oxide‐coated soda‐lime glass substrates (Nanoscribe, Germany). To provide sample adhesion, the substrates were silanized using 3‐(trimethoxysilyl)propyl methacrylate before printing. After the printing process, the samples were developed for 2 h in propylene glycol methyl ether acetate (≥99%, Carl Roth) and for another 24 h in acetone (ROTISOLV, ≥99.9%, Carl Roth), and thereafter dried in air.

### Filling of the Polymer Casts

The samples were evacuated in a desiccator using a membrane pump and flushed with helium gas (ALPHAGAZ 1 He, 99.999%, Air Liquide). The process was repeated 3 times. Afterward, the samples were kept in a small plastic flow box providing a helium environment. The volume flow of helium was ≈0.5 L min^−1^. After 15 min, the glass slurry (L40, Glassomer) was filled into the funnel of the structures via a 0.5 × 20 mm cannula. For illustration purposes, blue dye (Disperse Blue 134, Sigma‐Aldrich) was added to the glass slurry. The syringe containing the glass slurry was vortexed at 3000 rpm for 1 min before usage. The samples were exposed to ambient conditions as soon as the funnels were filled. After 90 min, the polymer casts were completely filled and the glass slurry was UV‐cured for 60 min. The buffer volume at the top of the polymer cast was clipped with pliers.

### Thermal Debinding and Sintering

For the heat treatment, the samples were transferred to sapphire substrates and put in a tube furnace (STF15/180, Carbolite Gero). The debinding process was conducted in air at ambient pressure as described in previous work.^[^ ^21^ ^]^ The sintering process was conducted under vacuum also along the lines of previous work,^[^ ^21^ ^]^ but with a reduced maximum temperature and holding time of 1225 °C and 10 min, respectively.

### Image Acquisition and Optical‐Image Cross‐Correlation

The image acquisition process was based on a setup already described previously.^[^ ^9^ ^]^ The microstructure samples were glued (UHU PLAST SPECIAL, UHU) onto a piezoactuator to provide a time‐harmonic axial excitation (PICMA Chip Actuator, Physik Instrumente, Germany). This excitation was synchronized to the stroboscopic illumination via two infrared light‐emitting diodes (850 nm center wavelength, Vishay, VSLY 3850), with a beat frequency of 1 Hz to provide slow‐motion videos of the high‐frequency motion of the microstructures. The samples were imaged from the side and the top with a 2.5× (Epiplan Neofluar 2.5×/0.075, Zeiss) and a 10× (Epilan 10×/0.20, Zeiss) microscope objective lens, respectively. The frequency‐dependent excitation amplitude was measured on the sample's bottom plate with a 25× microscope objective lens (Plan L 25×/0.40, Leitz Wetzlar). Optical images were acquired with a frame rate of 20 frames s^−1^ using two CMOS black/white cameras (BFLY‐PGE‐50S5M‐C and BFLY‐PGE31S4M‐C, FLIR Systems). Image cross‐correlation was performed with an open‐access software package.^[36^^]^

## Conflict of Interest

The authors declare no conflict of interest.

## Supporting information

Supporting Information

Supplemental Video 1

## Data Availability

The data that support the findings of this study are openly available in the KITopen repository at https://doi.org/10.5445/IR/1000135066.
