# Supplementary material for: Fused‐Silica 3D Chiral Metamaterials via Helium‐Assisted Microcasting Supporting Topologically Protected Twist Edge Resonances with High Mechanical Quality Factors
Source: Adv Mater. 2021 Aug 16;33(40):2103205. doi: 10.1002/adma.202103205 (PMC11468693; doi:10.1002/adma.202103205)
Supplement: Supplementary file 1 — Supporting Information [file ADMA-33-2103205-s001.pdf]

© 2021 Wiley-VCH GmbH

ADVANCED MATERIALS

Supporting Information

for *Adv. Mater.,* DOI: 10.1002/adma.202103205

Fused-Silica 3D Chiral Metamaterials via Helium-Assisted Microcasting Supporting Topologically Protected Twist Edge Resonances with High Mechanical Quality Factors

Julian Köpfler, Tobias Frenzel, Jörg Schmalian, and Martin Wegener**

Supporting Information

Fused-silica 3D chiral metamaterials via helium-assisted micro-casting supporting topologically protected twist edge resonances with high mechanical quality factors

Julian Köpfler, Tobias Frenzel, Jörg Schmalian, and Martin Wegener**

Geometrical Dimensions of the 3D Microstructure

Table S1. Geometrical parameters of the 3D microstructure (cf. Fig. 1).

Parameter	Value [a]	Description
l	$505\ \mu m$	Cube cell size
d	$0.0571 \cdot l$	Extruded depth of ring and plate elements
b_1	$0.0315 \cdot l$	Ring connector thickness
b_2	$0.0960 \cdot l$	Plate connector thickness
δ_1	$19.05°$	Ring connector angle of type 1 cubic cell
δ_2	$6.68°$	Ring connector angle of type 2 cubic cell
r_1	$0.3190 \cdot l$	Ring inner diameter
r_2	$0.3950 \cdot l$	Ring outer diameter
h	$0.9 \cdot l$	Micro-mirror height

[a] The values of targeted and manufactured 3D microstructures are consistent up to ±5%.

Micro Tuning-Forks

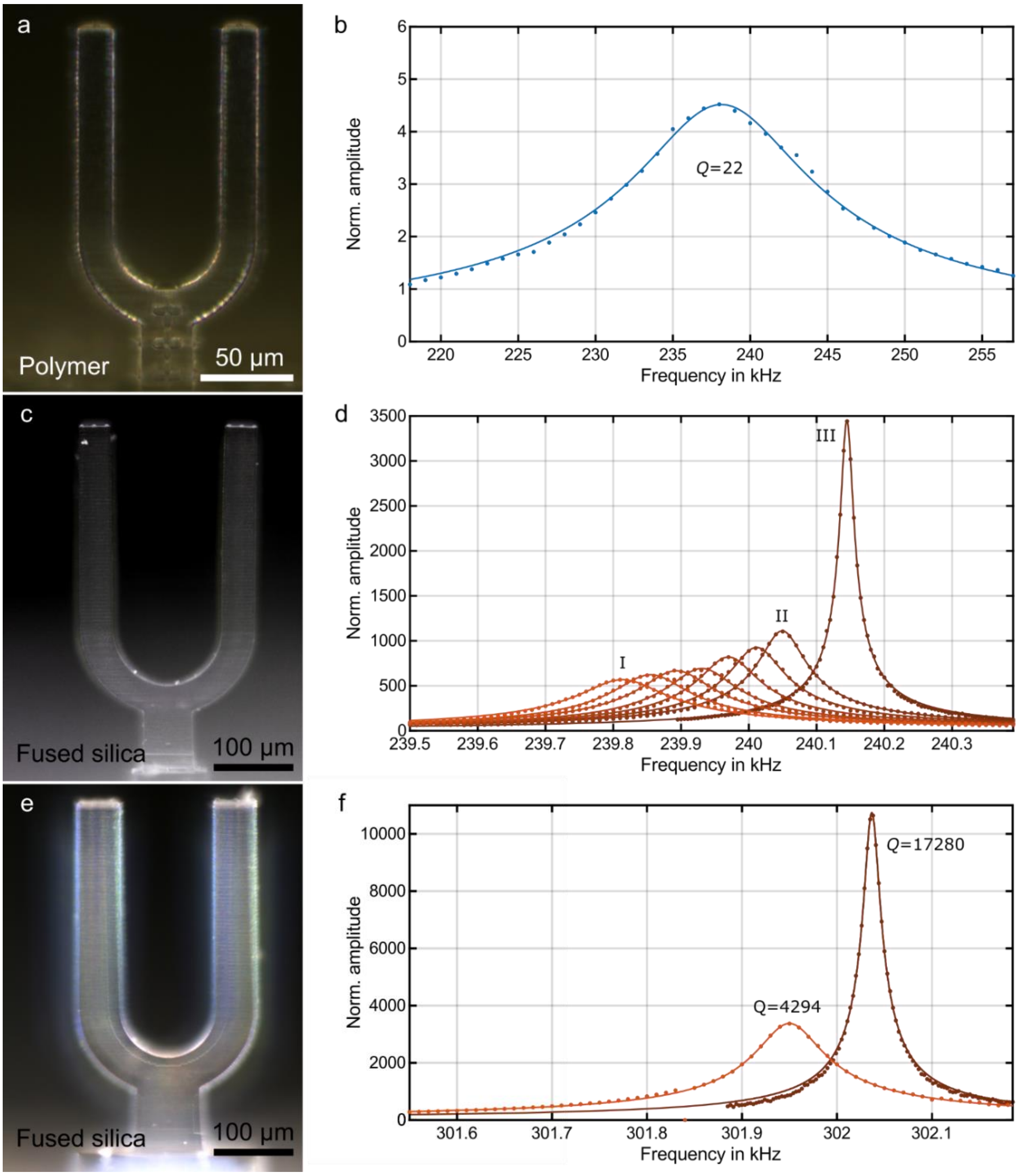

Figure S1. (a) Optical micrograph of a polymer micro tuning-fork fabricated by 3D nanoprinting and (b) measured amplitudes near the resonance frequency, normalized to the vertical excitation amplitude determined at the bottom plate of the structure. The quality factor of the resonance obtained via a Lorentzian fit of the squared amplitudes is $Q \approx 22$. (c) Optical micrograph of a fused-silica micro tuning-fork fabricated by helium-assisted micro-casting. (d) Corresponding resonance curves for 3 to 0 bar above ambient pressure (I to II), in steps of 0.5 bar, and under vacuum (III). By decreasing the air pressure, the quality factor increases from $Q \approx 1882$ (I) to $Q \approx 3582$ (II), and finally to $Q \approx 12450$ (III), indicating that air damping limits the quality factor. Comparing the resonance frequency with corresponding finite-element method (FEM) calculations reveals a Young's modulus of 70.8(±2.7) GPa, assuming a mass density of $\rho = 2.2$ g cm^{-3} and a Poisson's ratio of 0.17.[34,35] (e) For a different tuning fork design, the quality factor of the resonances (f) even reach values of $Q \approx 4294$ under ambient conditions and $Q \approx 17280$ in vacuum.

Displacement Data and Mode Shape in Top-view

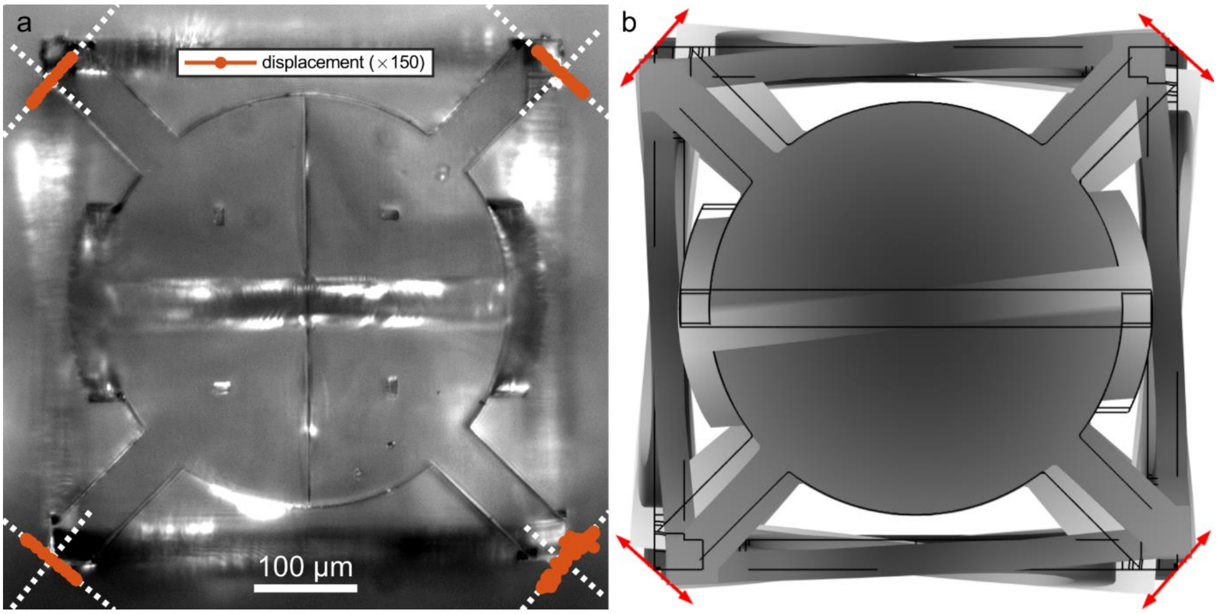

Figure S2. (a) Top-view optical micrograph of the fused-silica metamaterial-beam with tracked displacements for the top-end resonance frequency at 277.85 kHz at the four corners of the top plate supporting the micro-mirror. The displacements are scaled by a factor of 150 for better visibility. The azimuthal motion of the corners indicates a rotation of the top plate around its center of mass. (b) Corresponding linear-elastic finite-elements calculations reveal consistent displacements (red arrows) for the top-end resonance.

Video S1. See additionally submitted video file. The slow-motion video was acquired as described in the Experimental Section. It shows the fused-silica micro tuning-fork depicted in Figure S1c actuated at its resonance frequency (fundamental antisymmetric mode) with comparatively high vertical excitation amplitude (see scaled arrow at the bottom).

Role of Dissipation on the System of two Chirally Coupled Su-Schrieffer-Heeger Models

The topological characteristics of the system of two chirally coupled Su-Schrieffer-Heeger (SSH) models, which is the basis of the fabricated fused-silica microstructure, has been discussed thoroughly in our previous work.[25] However, the discussion did not incorporate dissipation, which is unavoidable and thus relevant for real world systems, e.g., due to the influence of material damping and air damping, as pointed out in our current work. In the following, we verify that distinct topologically trivial and nontrivial phases, as well as robust edge modes, are still guaranteed in the presence of dissipation.

We start with the known 4×4 wavenumber-dependent dynamical matrix $\widehat{D}$ of the two coupled SSH models, derived from a mass-spring model describing the 1D infinite diatomic chain of chiral 3D unit cells:[25]

$$\widehat{D}(k) = \begin{pmatrix} D_1 + D_2 & -D_1 - D_2 e^{-ika} & A_1 + A_2 & -A_1 - A_2 e^{-ika} \\ -D_1 - D_2 e^{ika} & D_1 + D_2 & -A_1 - A_2 e^{ika} & A_1 + A_2 \\ A_1 + A_2 & -A_1 - A_2 e^{-ika} & B_1 + B_2 & -B_1 - B_2 e^{-ika} \\ -A_1 - A_2 e^{ika} & A_1 + A_2 & -B_1 - B_2 e^{ika} & B_1 + B_2 \end{pmatrix}$$

and the corresponding eigenvalue problem:

$$\widehat{D}(k)\left|\boldsymbol{u}_{\mathrm{R}}^{(n)}(k)\right\rangle = \omega_n^2(k)\left|\boldsymbol{u}_{\mathrm{R}}^{(n)}(k)\right\rangle, \qquad \text{with} \qquad \left|\boldsymbol{u}_{\mathrm{R}}^{(n)}(k)\right\rangle = \begin{pmatrix} u_{\mathrm{a}}^{(n)}(k) \\ u_{\mathrm{b}}^{(n)}(k) \\ \varphi_{\mathrm{a}}^{(n)}(k) \\ \varphi_{\mathrm{b}}^{(n)}(k) \end{pmatrix}.$$

Here, a is the lattice constant, k is the wavenumber, and ω_n is the angular eigenfrequency of band n. The corresponding right eigenvector $\left|\boldsymbol{u}_{\mathrm{R}}^{(n)}(k)\right\rangle$ contains the complex amplitudes of the longitudinal and rotational degrees of freedom (DOF), $u^{(n)}(k)$ and $\varphi^{(n)}(k)$, respectively, at the two sublattice sites a and b. The four DOF are coupled via the intracell (intercell) longitudinal, torsional, and chiral spring constants, $D_1(D_2)$, $B_1(B_2)$, and $A_1(A_2)$, respectively. As a known prerequisite for the quantization of the Zak phase,[25] equal masses and moments of inertia at the two sublattice sites are assumed and thus can be absorbed by renormalization of the spring constants in the dynamical matrix.

To include damping, we assume linear viscoelastic behaviour of the constituent material of the microstructure, e.g., polymer or fused silica. In our experiments, the material is subject to a time-harmonic strain $\varepsilon(t) = \varepsilon_\omega e^{\mathrm{i}\omega t}$ with a fixed angular frequency ω, such that the stress σ can be described as[38]

$$\sigma_\omega = (E'_\omega + \mathrm{i}E''_\omega)\varepsilon_\omega.$$

Here, E'_ω and E''_ω are the real and imaginary part of the complex Young's modulus $E_\omega = E'_\omega + \mathrm{i}E''_\omega$, respectively. This ansatz can easily be understood using the standard Kelvin-Voigt model.[38] There, the stress-strain relationship is just the sum of a purely linear elastic contribution with a Young's modulus of E_0 and a viscous contribution with viscosity η

$$\sigma(t) = E_0\varepsilon(t) + \eta\dot{\varepsilon}(t).$$

Using again a time-harmonic strain $\varepsilon(t) = \varepsilon_\omega e^{\mathrm{i}\omega t}$ and stress $\sigma(t) = \sigma_\omega e^{\mathrm{i}\omega t}$, this results in

$$\sigma(t) = (E_0 + \mathrm{i}\eta\omega)\varepsilon_\omega$$

such that the real part and imaginary part of the complex Young's modulus are directly given by $E'_\omega = E_0$ and $E''_\omega = \eta\omega$, respectively. As we reduce the continuum description to a simple mass-and-spring model, the usage of a complex Young's modulus is equivalent to the usage of complex spring constants.

Thus, we allow the six different spring constants in the dynamical matrix $\widehat{D}(k)$ to be complex-valued, rendering the eigenvalue problem non-Hermitian. As a consequence, the right eigenvectors no longer form an orthogonal set. However, right eigenvectors continue to have orthogonality relations with the left eigenvectors $\left\langle \boldsymbol{u}_\mathrm{L}^{(n)}(k)\right|$ of the eigenvalue problem defined as:

$$\left\langle \boldsymbol{u}_\mathrm{L}^{(n)}(k)\right|\widehat{D}(k) = \omega_n^2(k)\left\langle \boldsymbol{u}_\mathrm{L}^{(n)}(k)\right|.$$

As long as the eigenvalues are non-degenerate, each left eigenvector is orthogonal to all right eigenvectors except for its corresponding one, and vice versa. This allows to generalize the definition of the Zak phase $\gamma^{(n)}$ for non-Hermitian eigenvalue problems as[29-31]

$$\gamma^{(n)} = \mathrm{i}\int_{-\pi/a}^{\pi/a}\left\langle \boldsymbol{u}_\mathrm{L}^{(n)}(k)\middle|\partial_k\,\boldsymbol{u}_\mathrm{R}^{(n)}(k)\right\rangle \mathrm{d}k.$$

To calculate the Zak phase numerically for a discretized version of the first Brillouin zone with N distinct wavenumbers k_i, it is favorable to directly calculate the phases between neighboring eigenvectors and sum them up over the first Brillouin zone to form a closed loop:

$$\begin{aligned}\gamma^{(n)} &= -\arg\left[\left\langle \boldsymbol{u}_\mathrm{L}^{(n)}(k_1)\middle|\boldsymbol{u}_\mathrm{R}^{(n)}(k_2)\right\rangle\left\langle \boldsymbol{u}_\mathrm{L}^{(n)}(k_2)\middle|\boldsymbol{u}_\mathrm{R}^{(n)}(k_3)\right\rangle \dots \left\langle \boldsymbol{u}_\mathrm{L}^{(n)}(k_{N-1})\middle|\boldsymbol{u}_\mathrm{R}^{(n)}(k_N)\right\rangle\right] \\ &= -\mathrm{Im}\left[\sum_{n=1}^{N-1}\ln\left[\left\langle \boldsymbol{u}_\mathrm{L}^{(n)}(k_n)\middle|\boldsymbol{u}_\mathrm{R}^{(n)}(k_{n+1})\right\rangle\right]\right].\end{aligned}$$

Here, the left and right eigenvectors are normalized according to

$$\left\langle \boldsymbol{u}_\mathrm{L}^{(m)}(k_i)\middle|\boldsymbol{u}_\mathrm{R}^{(n)}(k_i)\right\rangle = \delta_{mn}.$$

As a phase convention, the first component of the left eigenvectors for each wavenumber k was set to be real positive. To calculate the band structure and Zak phases of the individual bands for the described mass-spring model with damping, we use the known spring constants[25] and add an imaginary part of 5% to each of them, i.e., $D_1 \rightarrow D_1(1 + 0.05\mathrm{i})$. Along this line, we simulate the case of a relatively large material damping, as expected for a polymer (with a material quality factor of $Q \approx 20$) as a constituent material. The calculated band structure and Zak phases of the individual bands, the resulting edge modes, and an exemplary eigenvector evolution along the first Brillouin zone, are shown in **Figure S3**. The calculations verify that the Zak phases of the individual bands stay quantized in the presence of damping, and the existence of robust edge states is still guaranteed. We note that this holds also true for cases where the imaginary parts of the complex spring constants are not all fixed to the same ratio of the associated real parts, but rather chosen arbitrarily (not depicted), as long as the band gap stays opened for the chosen parameter configuration.

To summarize, the sharp distinction between topologically trivial and non-trivial states is not washed out by dissipative effects. Merely the location of the phase boundary between both regimes is altered. For example, for a single SSH chain, where the transition usually occurs at $D_1 = D_2$, we now find the criterion $|D_1| = |D_2|$.

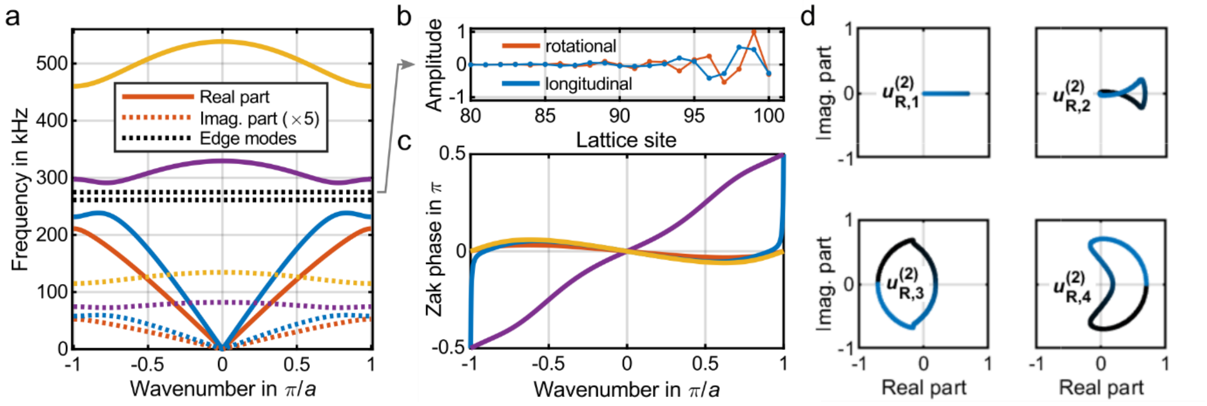

Figure S3. (a) Band structure of the mass-spring model of two coupled SSH models with real parts of the eigenfrequencies (solid lines) and associated imaginary parts (colored dotted lines) due to damping. Modes (black dotted lines) emerge in the band gap for a corresponding finite mass-spring model with 100 lattice sites, having one end fixed and the other end with an additional mass. (b) Both the rotational and longitudinal amplitudes of these modes (here exemplary the upper one) are located at the boundary, as expected. (c) Evolution of the Zak phases of the bands along the first Brillouin zone. The Zak phases are quantized (0 or $\pm\pi$) as for the case without damping, with two topologically trivial (red, yellow), and two non-trivial (blue, purple) bands. (d) Exemplary contours of the four complex eigenvector components of the blue band from $k = -\pi/a$ to $+\pi/a$ (gradually from dark to light blue), used to calculate the Zak phase evolution along the Brillouin zone.

Finite-Element-Method (FEM) Calculations

Table S2. Parameters and boundary conditions of the finite-element-method (FEM) calculations shown in Figures 3 and 4.

Type of FEM calculations	Parameter	Value
Eigenmodes/resonance calculations	COMSOL mesh size option	Extra fine
	Maximum element size	0.28 [a)]
	Minimum element size	0.012 [a)]
	Maximum element growth rate	1.35
	Curve factor	0.3
	Resolution of narrow regions	0.85
	Bottom plate boundary condition for eigenfrequencies/eigenmodes	Prescribed displacement $\boldsymbol{u}_0 = (0,0,0)$
	Bottom plate boundary condition for frequency domain/resonance	Prescribed displacement $\boldsymbol{u}_0 = (0,0,w_0)$
Band structure calculations	COMSOL mesh size option	Coarse
	Maximum element size	0.30 [a)]
	Minimum element size	0.056 [a)]
	Maximum element growth rate	1.6
	Curve factor	0.7
	Resolution of narrow regions	0.4
	Periodic boundary condition	Floquet periodicity $\boldsymbol{k}_F = (0,0,k)$

[a)] In units of the cube cell size l.

References

[1] T. Baldacchini, Three-dimensional Microfabrication Using Two-photon Polymerization: Fundamentals, Technology and Applications, Elsevier GmbH, Amsterdam, 2015

[2] V. Hahn, F. Mayer, M. Thiel, M. Wegener, *Opt. Photonics News* **2019**, 30, 28

[3] M. Malinauskas, H. Gilbergs, A. Žukauskas, V. Purlys, D. Paipulas, R. Gadonas, *J. Opt.* **2010**, 12, 035204

[4] T. Gissibl, S. Thiele, A. Herkommer, H. Giessen, *Nat. Photonics* **2016**, 10, 554

[5] H. E. Williams, D. J. Freppon, S. M. Kuebler, R. C. Rumpf, M. A. Melino, *Opt. Express* **2011**, 19, 22910

[6] V. Hahn, S. Kalt, G. M. Sridharan, M.Wegener, S. Bhattacharya, *Opt. Express* **2018**, 26, 33148-33157

[7] T. Bückmann, N. Stenger, M. Kadic, J. Kaschke, A. Frölich, T. Kennerknecht, C. Eberl, M. Thiel, M. Wegener, *Adv. Mater.* **2012** 24, 2710

[8] J. Bauer, L. R. Meza, T. A. Schaedler, R. Schwaiger, X. Zheng, L.Valdevit, *Adv. Mater.* **2017**, 29, 1701850

[9] T. Frenzel, J. Köpfler, E. Jung, M. Kadic, M. Wegener, *Nat. Commun.* **2019**, 10, 3384

[10] E. D. Lemma, B. Spagnolo, M. De Vittorio, F. Pisanello, *Trends Biotechnol.* **2019**, 37, 358

[11] M. Hippler, E. D. Lemma, S. Bertels, E. Blasco, C. Barner-Kowollik, M. Wegener, M. Bastmeyer, *Adv. Mater.* **2019**, 31, 1808110

[12] T. Limongi, L. Brigo, L. Tirinato, F. Pagliari, A. Gandin, P. Contessotto, A. Giugni, G. Brusatin, *Biomed. Mater.* **2021**, 16, 035013

[13] D. B. Phillips, G. M. Gibson, R. Bowman, M. J. Padgett, S. Hanna, D. M. Carberry, M. J. Miles, S. H. Simpson, *Opt. Express* **2012**, 20, 29679

[14] G. Adam, A. Benouhiba, K. Rabenorosoa, C. Clévy, D. J. Cappelleri, *Adv. Intell. Syst.* **2021**, 2000216

[15] A. I. Bunea, D. Martella, S. Nocentini, C. Parmeggiani, R. Taboryski, D. S. Wiersma, *Adv. Intell. Syst.* **2021**, 2000256

[16] X. Liu, M. Wei, Q. Wang, Y. Tian, J. Han, H. Gu, H. Ding, Q. Chen, K. Zhou, Z. Gu, *Adv. Mater.* **2021**, 33, 2100332

[17] P. Patimisco, G. Scamarcio, F. K. Tittel,V. Spagnolo, *Sensors* **2014**, 14, 6165

[18] P. Patimisco, A. Sampaolo, V. Mackowiak, H. Rossmadl, A. Cable, F. K. Tittel, V. Spagnolo, *IEEE. Trans. Ultrason. Ferroelectr. Freq. Control* **2018**, 65, 1951

[19] F. Kotz, K. Arnold, W. Bauer, D. Schild, N. Keller, K. Sachsenheimer, T. M. Nargang, C. Richter, D. Helmer, B. E. Rapp, *Nature* **2017**, 544, 337

[20] F. Kotz, N. Schneider, A. Striegel, A. Wolfschläger, N. Keller, M. Worgull, W. Bauer, D. Schild, M. Milich, C. Greiner, D. Helmer, B. E. Rapp, *Adv. Mater.* **2018**, 30, 1707100

[21] F. Kotz, P. Risch, K. Arnol, S. Sevim, J. Puigmartí-Luis, A. Quick, M. Thiel, A. Hrynevich, P. D. Dalton, D. Helmer, B. E. Rapp, *Nat. Commun.* **2019**, 10, 1439

[22] D. G. Moore, L. Barbera, K. Masania, A. R. Studart, *Nat. Mater.* **2020**, 19, 212

[23] D. Zhang, X. Liu, J. Qiu, *Front. Optoelectron.* **2020**

[24] F. Kotz, A.S. Quick, P. Risch, T. Martin, T. Hoose, M. Thiel, D. Helmer, B. E. Rapp, *Adv. Mater.* **2021**, 33, 2006341

[25] J. Köpfler, T. Frenzel, M. Kadic, J. Schmalian, M. Wegener, *Phys. Rev. Appl.* **2019,** 11, 034059

[26] E. Prodan, C. Prodan, *Phys. Rev. Lett.* **2009** 103, 248101

[27] R. Süsstrunk, S. D. Huber, *Proc. Natl. Acad. Sci. U.S.A.* **2016** 113, E4767

[28] Y. Barlas, E. Prodan, *Phys. Rev. B* **2018** 98, 094310

[29] F. Zangeneh-Nejad, A. Alù, R. Fleury, *C. R. Phys.* **2020**, 467

[30] J. C. Garrison, E. M. Wright, *Phys. Lett. A* **1988** 128, 177

[31] K. Esaki, M. Sato, K. Hasebe, M. Kohmoto, *Phys. Rev. B* **2011** 84, 205128

[32] S. Lieu, *Phys. Rev. B* **2018** 97, 045106

[33] Y.-H. Park, K. C. Park, *J. Microelectromech. Syst.* **2004**, 13, 238

[34] N. P. Bansal, R. H. Doremus, Handbook of Glass Properties, Elsevier, Orlando, FL 2013

[35] M. Fukuhara, A. Sanpei, K. Shibuki, *J. Mater. Sci.* **1997**, 32, 1207

[36] C. Eberl, T. Thompson, D. Gianola, W. Sharpe Jr., K. Hemker, Digital image correlation and tracking. MatLabCentral, Mathworks file exchange server, FileID 12413, 2006

[37] T. Frenzel, J. Köpfler, A. Naber, M. Wegener, *Sci. Rep.* **2021** 11, 2304

[38] A. S. Nowick, B. S. Berry, Anelastic Relaxation in Crystalline Solids, Academic Press, New York, 1972
